# Tim-4 in Health and Disease: Friend or Foe?

**DOI:** 10.3389/fimmu.2020.00537

**Published:** 2020-04-02

**Authors:** Wen Liu, Liyun Xu, Xiaohong Liang, Xiaojun Liu, Yangbing Zhao, Chunhong Ma, Lifen Gao

**Affiliations:** ^1^Key Laboratory for Experimental Teratology of Ministry of Education, Shandong Provincial Key Laboratory of Infection and Immunology, Department of Immunology, School of Basic Medical Sciences, Shandong University, Jinan, China; ^2^Cell and Molecular Biology Laboratory, Zhoushan Hospital, Zhoushan, China; ^3^Center for Cellular Immunotherapies, University of Pennsylvania Cancer Center, Philadelphia, PA, United States; ^4^Department of Pathology and Laboratory Medicine, Perelman School of Medicine, University of Pennsylvania, Philadelphia, PA, United States

**Keywords:** Tim-4, macrophages, T cells, phosphatidylserine, immune regulation

## Abstract

T-cell immunoglobulin and mucin domain containing 4 (Tim-4) is a phosphatidylserine receptor and is selectively expressed on antigen presenting cells. Recently, Tim-4 was reported to be expressed on iNKT cells, B1 cells, and tumor cells, suggesting it has multiple biological functions. In this review, we mainly summarize the expression and regulation of Tim-4 in immune cells including T cells, macrophages, dendritic cells, NKT cells, B cells, and mast cells. The expression of Tim-4 in these cells implies that Tim-4 might participate in immune related diseases. Emerging evidence emphasizes a substantial role for Tim-4 in maintaining homeostasis by regulating various immune responses, including viral infection, allergy, autoimmunity, and tumor immunity. Here, we collectively evaluated the role of Tim-4 in health and diseases. This summary will be extremely useful to fully understand the function of Tim-4 in the pathogenesis of immune related diseases, which would provide novel clues for the diagnosis and treatment of diseases.

## Introduction

The T cell immunoglobulin domain and mucin domain (*Tim*) gene family is a relatively new gene family and was discovered in 2001 (1). Totally, the *Tim* family contains three members (*TIM-1, TIM-3, TIM-4*) in humans, and eight members (*Tim-1* to *Tim-8*) in mice. The *Tim* gene family is an attractive area of research due to its location on the mouse chromosome 11B1.1 or human chromosome 5q33.2, which is related to asthma, allergic diseases, and autoimmune diseases (2–4). Unlike other Tim molecules, Tim-4 is mainly expressed on antigen-presenting cells (APCs) but not on T cells (5). In addition, Tim-4, identified as a natural ligand of Tim-1, can modulate T cell proliferation, which is involved in the development of multiple immune diseases (6, 7).

Shortly after the discovery of the *Tim* gene family, in 2004, Shakhov et al. found a selectively downregulated gene named *SMUCKLER* (Spleen, Mucin-Containing Knockout of Lymphotoxin) in *LT*-α and *LT*-β deficient mice (8). This gene encodes a 60 kDa type I transmembrane protein including a signal peptide, a characteristic of immunoglobulin variable region-like (IgV) domain, a mucin-like region, a transmembrane region, and an intracellular tail region, which was later identified as Tim-4. The IgV-like domain contains a conserved Arginine-Glycine-Aspartic acid (RGD) motif, which can bind to integrin proteins. The mucin-like domain of Tim-4 is the longest of the Tim family members and is rich in threonine, serine, and proline residues. The intracellular region of Tim-4 contains about 42–77 amino acids, which is the most highly conserved region of mouse and human homologs. In particular, the intracellular domain of Tim-4 lacks tyrosine activation residues. Therefore, the expression profile and special structure of Tim-4 might indicate its intriguing role in health and diseases.

## Introduction to Tim-4

Tim-4 is highly expressed in peripheral lymphoid tissues, such as the tonsils, thymus, spleen, lymph nodes (LNs), and Peyer's nodule but is poorly expressed in the lung, liver, and kidney tissues (5, 9, 10). It has been confirmed that Tim-4 is selectively expressed in macrophages, mature dendritic cells (DCs), peritoneal B1 cells, and invariant natural killer T (iNKT) cells (9, 11). Furthermore, the levels of Tim-4 expression can be regulated by different stimulators, such as lipopolysaccharide (LPS), cholera toxins, cytokines, Concanavalin A (ConA), and danger associated molecular patterns (DAMPs) (12–16). However, Tim-4 expression can also be downregulated under some conditions. It is reported that probiotics such as *Bifidobacterium infantis* reduced Tim-4 expression in DCs by inhibiting its transcription factor, STAT6 (17). Vitamin D (VitD) might repress the Tim-4 gene transcription and expression *via* VitD receptor (18). In addition to expression in immune cells, Tim-4 was also found to be ectopically expressed in tumor cells, including lung cancer (19), colorectal cancer (20), juvenile xanthogranuloma, tissue sarcomas, Langerhans cell sarcoma, and parapharyngeal liposarcoma (21).

Specific microenvironment modulates Tim-4 expression. We observed that Tim-4 expression was relatively low in lung cancer cell lines, while its expression was increased in lung cancer tissues. Furthermore, Tim-4 expression was greatly enhanced by IL-6 or TGF-β, which were highly abundant in tumor microenvironment (19, 22, 23). Our studies also showed that microenvironment of non-alcohol fatty liver disease increased Tim-4 expression in liver tissues greatly, especially in macrophages (24). These data suggest that Tim-4 is inducible under specific disease conditions. However, the mechanisms governing Tim-4 expression remain largely elusive. Some evidences suggested that the histone acetyltransferase, p300, and STAT6 regulate the expression of Tim-4 in DCs upon stimulation by the cholera toxin (25). Recently, it was found that cigarette smoke extract (CSE) upregulated Tim-4 expression in immature DCs from murine bone marrow, and the upregulation of Tim-4 stimulated by CSE was inhibited by an ERK inhibitor but not by a p38 or JNK inhibitor (26). Further research is required to elucidate the mechanisms driving transcriptional and translational regulation of Tim-4.

Emerging evidences showed that Tim-4-Ig could bind to T cells, suggesting that Tim-4 receptors might exist on the surface of T cells. It was subsequently shown that Tim-4-Ig bound with CHO cells transfected with Tim-1, but not Tim-3 or Tim-4, confirming the interaction between Tim-4 and Tim-1 (5). In addition, Tim-4-Ig bound with activated T cells, which expressed high levels of Tim-1, and this binding was blocked by antibodies against Tim-1, suggesting that Tim-4 is indeed a natural ligand of Tim-1 (5). Another study showed that Tim-4-Ig showed high affinity for early and late apoptotic Jurkat cells, but not living cells (10). Subsequent results demonstrated that Tim-4 could bind to phosphatidylserine (PS) exposed on the surface of apoptotic cells. Therefore, Tim-4 was identified as another receptor of PS (27). Besides, they found that both Tim-1 and Tim-4 bound PS exposed on apoptotic cells or exosomes, which led to the realization that the Tim-1–Tim-4 interaction occurred through a PS bridge. Thus, the interaction between Tim-1 and Tim-4 is indirect (27). In addition, leukocyte mono-immunoglobulin (Ig)-like receptor 5 (LMIR5) also interacted with Tim-4, suggesting that Tim-4 is a possible ligand for LMIR5. Nevertheless, LMIR5 neither bound to PS nor affected Tim-4-mediated phagocytosis of apoptotic cells (28). Of course, there might be other unidentified receptors of Tim-4.

## Regulation of Tim-4 on Immune Cells

### T Lymphocytes

It is reported that Tim-4 mRNA is not expressed in T cells, however, Tim-4 displays modulation on T cells through its receptor. Rodriguez-Manzanet et al. found that the proliferation of T cells incubated with Tim-4-expressing CHO cells was significantly higher than that of control cells (29). Blockade with a Tim-4 antibody partially inhibited T cell proliferation induced by Tim-4-expressing CHO cells, suggesting that Tim-4 is involved in promoting T cell activation. CHO cells also express endogenous co-stimulatory molecules, and these molecules, in conjunction with Tim-4, may play a synergistic role in T cell activation (29). Interestingly, the regulation of Tim-4 on T cells has two sides. The dose of Tim-4 is critical for its effect on T cells. Higher doses of Tim-4-Ig promoted T cell proliferation and amplification *in vitro* and *in vivo*, while lower concentrations of Tim-4-Ig strongly inhibited T cell proliferation induced by anti-CD3 and anti-CD28 antibodies (5). Moreover, Tim-4 exerts bimodal functions depending on the activation status of T cells. It was reported that Tim-4 bound to naïve CD4^+^ T cells that did not express Tim-1, and Tim-4 inhibited naïve CD4^+^ T cell proliferation but not of pre-activated T cells (7). However, Cao et al. found that Tim-4 Fc could inhibit both naïve and pre-activated T-cell activation, proliferation and cytokine production *via* a Tim-1-independent pathway (30). These inconsistent findings might attribute to different experimental conditions. In addition, Ge et al. reported that Tim-4 could also bind Tim-3 on the surface of polarized T helper type 1 (Th1) cells to increase p300 phosphorylation in Th1 cells, which further increased the levels of Fas ligand in the cells and induced Th1 cell apoptosis (31). These data suggest that Tim-4 might regulate T cell activation by cross-linking different receptor, and unknown receptors on T cells are required to be identified.

It is well-known that the MAPK pathway is essential for T cell proliferation and differentiation (32–34). Tim-4 Ig could induce Akt and ERK phosphorylation of T cells by interacting with Tim-1. Tim-4-Ig induced Akt phosphorylation and increased Bcl-2 protein expression were involved in the protection of activated T cells from apoptotic cell death. However, the presence of Tim-4-Ig did not increase Bcl-xL protein expression (29). Thus, it is possible that Tim-4 promotes T cell proliferation by induction of cell division and anti-apoptotic signals. However, Tim-4-Fc inhibited ERK phosphorylation of T cells stimulated with coated anti-CD3 and anti-CD28 (7), suggesting that Tim-4-Fc could also inhibit the MAPK pathway even in pre-activated T cells expressing Tim-1. Cao et al. also demonstrated that Tim-4 Fc could inhibit the MAP kinase pathway in T cells. Tim-4 Fc not only inhibited T cell activation but also prevented the differentiation of Th17 cells, even blocked IL-17 production in Th17-polarized cultures (30). This study also showed that both IgV and mucin domains were required for Tim-4 mediated inhibition of T-cell activation and Th17 differentiation *via* the MAPK pathway (30).

### Macrophages

We reported that Tim-4 inhibited the expression of CD80, CD86, MHC-II and the production of TNF-α in LPS-treated macrophages (15). Tim-4 could also suppress nitric oxide (NO) production in LPS- or IFN-γ- activated macrophages (14). Furthermore, Tim-4 was shown to protect mice from ConA-induced liver injury or LPS-induced septic shock by inhibiting pro-inflammatory cytokines such as IL-6 and TNF-α (15, 35). Although Con A-induced liver injury is a T cell-mediated autoimmune hepatitis model (36), it has been found that Kupffer cells are involved in the initiation and propagation of liver damages (37), suggesting that Tim-4 expressed on macrophages might attenuate the priming and activation of T cell responses in Con A-induced hepatitis. Further analysis showed that Tim-4 inhibited cytokines and NO production *via* the NF-κB or Jak2/STAT1 signaling pathways (14). Taken together, we speculate that Tim-4 may play an inhibitory role in the course of macrophage activation.

Another important function of macrophages is phagocytosis, which plays an essential role in the elimination of apoptotic cells to maintain homeostasis. It is known that PS exposure on the cell surface is the “eat me” signal of apoptotic cells for engulfment and removal called efferocytosis (38). Tim-4 enhances Tyro3, Axl, and Mer receptors (TAMs)-stimulated efferocytosis in resident peritoneal macrophages, Kupffer cells, and CD169^+^ skin macrophages, but not in thioglycollate-elicited peritoneal macrophages or primary cultured microglia, which do not express Tim-4 (39). Both Kobayashi et al. and Miyanishi et al. identified Tim-4 as a PS receptor for the engulfment of apoptotic cells, and the phagocytosis of apoptotic thymocytes was decreased when peritoneal macrophages were pretreated with Tim-4-blocking antibody (10, 27). Santiago et al. found that crystal structures of Tim-4 had a metal-ion-dependent ligand binding site (MILIBS) for PS binding, and any amino acid deficiency or mutation in the MILIBS region of Tim-4 would lead to impaired phagocytosis (40). Tietjen et al. further clarified that the sensitivity of Tim-4 to recognize PS was related to the surface density of PS, as well as the composition and the fluidity of the membrane (41).

Park et al. explored the downstream pathway for the recognition of PS by Tim-4. They declared that Tim-4 was independent of the two known engulfment signaling pathways: the ELMO1/Dock180/Rac pathway and GULP-mediated pathway (42). In contrast, it was found that the transmembrane region of Tim-4 was dispensable for Tim-4-mediated engulfment, suggesting that the potential membrane molecule binding to the extracellular region of Tim-4 could coordinate the signaling together with Tim-4. As expected above, Flannagan et al. found that integrin proteins bound to Tim-4 as a co-receptor to induce the signal transduction cascade required for phagocytosis of apoptotic cells (43). Recently, Lee et al. identified Fibronectin (Fn1) as a novel Tim-4-associating protein, and found that Fn1 acted as a scaffold to form the complex composed of Tim-4 and integrin to mediate efferocytosis (44).

Interestingly, Tim-4 emerges as a key marker of resident macrophage in various tissues. Recruited macrophages do not express Tim-4, highlighting that Tim-4 is required for a specific population of macrophages. At steady state, Tim-4^+^ resident cardiac macrophages, which were LYVE1^+^MHC-II^low^CCR2^−^, maintained self-renewal despite low input of blood monocytes. Following myocardial infarction, macrophages promoted both injury and repair, while resident cardiac macrophages played a non-redundant, cardioprotective role by limiting adverse remodeling (45). In the intestine, tissue-resident macrophages were long lived and defined by Tim-4 and CD4 expression (46, 47). In lean, murine visceral adipose tissues, the majority of adipose tissue-resident F4/80^hi^ macrophages (ATM) expressed Tim-4, and embryonic-derived Tim-4^+^MHCII^low^ and Tim-4^+^MHCII^+^ ATM subsets were long-lived (48). Likewise, Tim-4 was essential for the maintenance of resident peritoneal macrophage homeostasis (9).

### Dendritic Cells

DCs are the most potent antigen-presenting cells to initiate a naïve T cell response. Yeung et al. (49) reported that the blockade of Tim-4 on the surface of DCs could promote the transformation of naïve CD4^+^ T cells to induced regulatory T cells (iTreg) in skin allografts both *in vitro* and *in vivo*. Li et al. (50) found that DC infiltration and Tim-4 expression were increased in hepatic warm ischemia reperfusion (IR) models, and Tim-4 blockade on DCs significantly attenuated hepatic injury and reduced the release of pro-inflammatory cytokines. They found that Tim-4 blockade inhibited Th2 cell differentiation and facilitated induced CD4^+^CD25^+^Foxp3^+^ iTreg generation through the IL-4/STAT6 signaling pathway *in vitro*. *In vivo* data showed that adoptive transfer of iTreg induced by Tim-4 blockade into IR mouse models remarkably attenuated liver injury. Therefore, Tim-4 on DCs played a critical role in mediating hepatic IR injury. In a model of allergy-induced colitis, it was found that Tim-4 expression on DCs was increased greatly (51). However, its role in colitis remains unknown. Collectively, these studies identify a key role for Tim-4 in immune response by regulating DCs, suggesting that Tim-4 might be an efficient target for the prevention of IR injury in the liver or other tissues.

Recently, Zhang et al. (52) found that the expression of Tim-4 was variable in the different subsets of DCs in skin and skin-draining LNs that were studied. Dermal CD207^+^ DCs and LNs resident CD207^−^CD4^+^ DCs highly expressed Tim-4. In Tim-4-deficient mice, it was found that loss of Tim-4 significantly upregulated the population of epidermal Langerhans cells and LNs resident CD207^−^CD4^+^ DCs, which was consistent with that in macrophages (53).

### Other Immune Cells

Previously, it was shown that Tim-4 was expressed on thymic iNKT cells, and its expression was increased upon iNKT cell migration to the LNs. However, Tim-4 was dispensable for the development and function of iNKT cell at steady state (11). Tim-4 identified proinflammatory B effector 1 (Be1) cells, and Tim-4^+^ Be1 cells could decrease B16-F10 growth and metastasis in an IFN-γ-dependent manner (54). Moreover, Tim-4^+^ Be1 cells could promote pro-inflammatory Th cell differentiation *in vivo* and increase IFN-γ production while decreasing IL-4, IL-10, and Foxp3 expression. Importantly, blockade of Tim-4 could promote allograft tolerance, which was dependent on Be1 cell expression of Tim-4 (54).

Tim-4 is involved in mast cell activation. Tim-4 cross-linking of Tim-1 enhanced cytokine production without affecting degranulation *via* signaling pathways downstream of FcεRI in mast cells (55). In addition, stimulation with Tim-4 Ig also induced LMIR5-mediated activation of mast cells (28). Though mast cells did not constitutively express Tim-4, flagellin could induce mast cells to express Tim-4 by increasing STAT6 phosphorylation (56).

## The Role of Tim-4 in Diseases

Since Tim-4 is closely related with immune regulation, it is likely that Tim-4 is involved in numerous diseases by affecting the immune system.

### Allergy

It is thought that misregulation of Th1/Th2 cells plays a critical role in the pathophysiology of asthma. Studies showed that several single nucleotide polymorphisms (SNPs) of Tim-1 were associated with asthma susceptibility in some populations (57, 58). In consideration of the associations between Tim-1 and Tim-4, McIntire et al. presented the hypothesis that several SNPs in *Tim-4* promoter might be associated with the susceptibility of asthma (1). Cai et al. reported an important relationship between the *Tim-4*−1419 G>A SNP and childhood asthma susceptibility in the Han population of China (59).

Cockroach allergen rBla g 7, the crucial factor in cockroach allergy, enhanced Tim-4 expression in DCs in a dose-dependent manner, suggesting that rBla g 7 challenged DCs induce Th2 polarization *via* Tim-4-, CD80-, and CD86-dependent mechanisms (60). Accumulating evidences showed that bacteria-associated factors were also linked to the development of allergies (61). Consistently, the simultaneous exposure to cholera toxin and peanuts resulted in an increase in Tim-4, MHC II, and co-stimulatory molecules expression in DCs, which could induce differentiation and activation of peanut-specific Th2 cells in the intestine. However, blocking the interaction between Tim-4 and Tim-1 with antibodies inhibited the allergic reaction, indicating that modulating Tim-4 function may prove to be an efficient treatment for DC-induced peanut allergy in the future (12). In allergic rhinitis patients, VitD deficiency may contribute to the pathogenesis by increasing the Tim-4 expression on DCs (18).

### Autoimmune Disease

Autoimmune disease is characterized by the impaired structure and function of multiple tissues. Many factors contribute to the development of autoimmunity, including the deficient removal of apoptotic cells (62). Consequences of inefficient clearance of apoptotic bodies include the onset of autoimmune diseases, such as rheumatoid arthritis (RA) and systemic lupus erythematosus (SLE) (63). Tim-4 mediates the clearance of apoptotic bodies by macrophages, and plays a pivotal role in autoimmune diseases (64).

We reported that *Tim-4* mRNA in PBMCs of SLE patients was significantly increased when compared to healthy controls. Additionally, Tim-4 was positively correlated with SLE (65). Though the correlation between two *Tim-4* SNPs rs6874202, rs62382402 and SLE susceptibility was not found in a Chinese Han population, the GG genotype of the *Tim-4* gene at−1419 site might be associated with the disease activity of SLE (66). Miyanishi et al. demonstrated that mice lacking *Tim-4* or *MFG-E8* (Milk Fat Globule EGF Factor VIII) developed little or no autoimmunity. In contrast, mice lacking both *Tim-4* and *MFG-E8* produced a high level of autoantibodies. Moreover, this process was accelerated by the administration of an anti-TNF-α antibody or pristane, a reagent that provokes type I IFN production (67). However, Rodriguez-Manzanet et al. found a significant development of auto-antibodies in Tim-4-deficiency mice on the B6 background (68). Although all mice were kept under sterile conditions, environmental factors might contribute to discrepancies in the experimental outcomes (69).

The associations between *Tim-4* SNP rs7700944 and RA susceptibility were described in the Chinese Han and Hui populations, Zahedan, southeast Iran, and Egyptian population (70–72). However, in collagen-induced arthritis (CIA), Tim-4 displayed dual function in the induction and effector phases. In the induction phase, anti-Tim-4 mAb exacerbated the development of CIA, while the arthritis scores and proinflammatory cytokines were reduced in anti-Tim-4 treated mice at effector phase (6). In the induction of experimental autoimmune encephalomyelitis (EAE) model, anti-Tim-4 treatment greatly ameliorated the clinical feature of EAE (7). Thus, the role of Tim-4 in autoimmunity is complicated, and reasonable therapy plans are being developed by targeting Tim-4.

### Tumor

Baghdadi et al. found that Tim-4 was highly expressed on tumor related macrophages and DCs stimulated by DAMPs such as HMGB1, heat shock protein 90, monosodium urate, S100A8, or ATP (apyrase) secreted from dying or stressed tumor cells induced by chemotherapy. Upon ingestion of apoptotic B16-F10 tumor cells triggered by chemotherapy, Tim-4-AMPKα1 interaction activated autophagy in macrophages, which could mediate degradation of ingested tumor cells, resulting in reduced antigen presentation and damaged cytotoxicity T lymphocyte responses (16). Xu et al. reported that glioma-derived macrophages, expressing high levels of Tim-4, contributed to tumor tolerance by phagocytizing T cells following PS exposure (73). Data from *Tim-4* transgenic mice indeed demonstrated that overexpression of Tim-4 on APCs resulted in decreased secondary T cell responses and decreased Ag-specific T cell numbers (74), thus Tim-4 played a negative role in antitumor immunity by inducing tumor immune tolerance. Consistently, a unique subset of CD163^+^Tim-4^+^ resident omental macrophages was defined to be responsible for metastatic spread of ovarian cancer cells recently. Moreover, CD163^+^Tim-4^+^ tissue-resident macrophages promoted the circulating stem cell-like phenotype of ovarian cancer cells (75). Thus, the therapeutic manipulation of Tim-4 may provide a novel strategy to enhance antitumor immunity and boost cancer chemotherapy.

Experimental models of tumor revealed that combined treatment with anti-Tim-3 and anti-Tim-4 mAbs increased the efficacy of cancer vaccines (76). In accordance, Jinushi et al. revealed that a secreted PS-binding protein MFG-E8 blockade also favored the establishment of an immunogenic tumor microenvironment (77). Consistent with this issue, novel monoclonal antibodies targeting Tim-4 could enhance the curative effects of vaccinations against melanomas in B16 mice. Blocking Tim-4 increased vaccine-induced antitumor responses against irradiated B16 melanoma mice by increasing the numbers of CD8^+^T cells and effector functions (78). Interestingly, a recent study indicated that an siRNA targeting the FG–CC′ loop could enhance the therapeutic effects of DC vaccines against gastric cancer, suggesting that targeting the FG–CC′ loop in Tim-4 might be helpful in the development of novel immunotherapeutic treatments (79).

Recently, ectopic expression of Tim-4 in tumor tissues was discovered, and Tim-4 was shown to act as an oncogene. We found that Tim-4 expression was significantly higher in non-small-cell lung cancer (NSCLC) tissues, and overexpression of Tim-4 was associated with adverse prognosis. Mechanistically, overexpression of Tim-4 promoted lung cancer cell growth and proliferation, depending on its Arg-Gly-Asp (RGD) motif of IgV domain. Furthermore, Tim-4 could interact with αvβ3 integrin through RGD motif (19). In addition, we found that Tim-4 also promoted migration, invasion, and epithelial mesenchymal transformation of NSCLC, which was responsible for IL-6 mediated metastasis of lung cancer (80). Li et al. found that Tim-4 expression was also significantly increased in glioma tissues. In the human glioma cell line LN-18 cells, Tim-4 was shown to promote growth, suppress apoptosis, and enhance clonogenicity (81). Tim-4 was upregulated in colorectal cancer and promoted the growth of colorectal cancer by activating angiogenesis and recruiting tumor-associated macrophages *via* the PI3K/AKT/mTOR signaling pathways (20).

The above data indicate that targeting Tim-4 might simultaneously reverse immune tolerance and suppress tumor cell growth and migration, which would provide a promising tool for tumor therapy. Therefore, it is required to specifically evaluate immune regulatory drugs such as Tim-4 inhibitors to define their anti-tumorigenic actions during tumorigenesis (82, 83). However, no effective Tim-4 inhibitors have been developed to date.

### Pathogen Infection

More recent studies revealed that Tim family members played critical roles in enveloped viral infections possibly by interacting with virion-associated PS. It was found that various enveloped viruses could expose PS on their membranes. Tim-1 and multiple enveloped viruses were dependent on PS as demonstrated by blocking liposome and Tim-1 mutagenesis experiments, and the function of additional PS receptors especially hTim-4 paralleled with hTim-1 (10). Similar to Tim-1, residues in the PS binding pocket of murine and human Tim-4 were found to be important for Ebola virus (EBOV) entry, and additional Tim-4-specific residues were also found to impact virion binding and internalization, which provided a greater understanding of the interaction between Tim-4 and EBOV virions (84). Interestingly, Tim-EBOV interactions were mechanically comparable to adhesion molecule-ligand interactions and the Tim-4-PS interaction was more resistant to mechanical force than the Tim-1-PS interaction (85). Exosomes from multiple sources increased HIV-1 entry into T cells and macrophages, and viral entry was potently blocked with anti-Tim-4 antibodies (86). In conclusion, hTim-4 and hTim-1 significantly contributed to infection by diverse families of enveloped viruses, thereby making them an attractive therapeutic target for the intervention of viral infections in the future (87).

In addition, Tim-4 contributed to efficient cell-to-cell spread by Listeria monocytogene (Lm) in macrophages *in vitro* and growth of these bacteria was impaired in *Tim-4*^−/−^ mice. Thus, Lm promoted its dissemination in a host by exploiting efferocytosis. This study suggests that PS-targeted therapeutics may be useful in the fight against infections by Lm and other bacteria that utilize similar strategies of cell-to-cell spread during infection (88). Also, we believe that antagonists of Tim-4 would be useful to block the spread of bacterial or viral infection.

### Chronic Metabolic Disease

SNP site is the main content of genetic polymorphism study. Recently, genome-wide association studies or meta-analysis from different laboratories showed that some SNPs of *Tim-4* gene were closely related to lipid metabolism. It was reported that *TIMD4* SNP (rs1501908) was closely related to low density lipoprotein cholesterol (LDL-C) in populations from Korea and Europe (89, 90). It was also shown to be negatively correlated with low density lipoprotein (LDL) and total cholesterol (TC) and positively correlated with high density lipoprotein in a Chinese population (91). SNPs in the promoter region of *TIMD4* rs6882076 was related to LDL, triglyceride and TC (92), and rs1553318 was also related to hyperlipidemia (93).

The level of Tim-4 mRNA expression in peripheral blood of patients with Type 2 diabetes mellitus was significantly increased and negatively correlated with LDL (94). The latest report found that blocking Tim-4 could aggravate atherosclerosis in *ldlr*^−/−^ mice (95), and the expression of Tim-4 mRNA was increased in liver tissues of rats on a high fat diet (96). Recently, we found that *Tim-4* knockout aggravated methionine and choline-deficient diet induced NAFLD, suggesting the protective role of Tim-4 in fatty liver disease. Furthermore, we revealed that Tim-4 promoted AMPKα phosphorylation depending on LKB1. Tim-4/LKB1/AMPKα interaction triggered autophagy and inhibited NLRP3 inflammasome activation, which might be responsible for NAFLD progress (24). However, we could not exclude whether Tim-4 was involved in lipid metabolism, a possibility that warrants further investigation.

## Soluble Tim-4

Tim-4 protein can be sheared into soluble Tim-4 (sTim-4) molecule by A Disintegrin and Metalloprotease 10 (ADAM 10) and ADAM 17, without affecting their binding to PS (97). sTim-4 could be detected in serum or bodily fluids, and the changes of sTim-4 might indicate pathological conditions. Following a stroke, we found that sTim-4 was significantly increased by days 2 and 5. Furthermore, stroke severity was positively correlated with sTim-4 levels in plasma. Up-regulated sTim-4 in plasma might act as a prognostic biomarker of ischemic stroke (98). In addition, serum levels of sTim-4 were significantly increased in ankylosing spondylitis patients, which was positively correlated with TNF-α levels and Bath ankylosing spondylitis disease activity index (99). However, we did not confirm the role of sTim-4 *in vivo*. We speculate that sTim-4 might act as an antagonist of membrane surface Tim-4, which requires further investigation.

## Discussion

**Figure 1:**
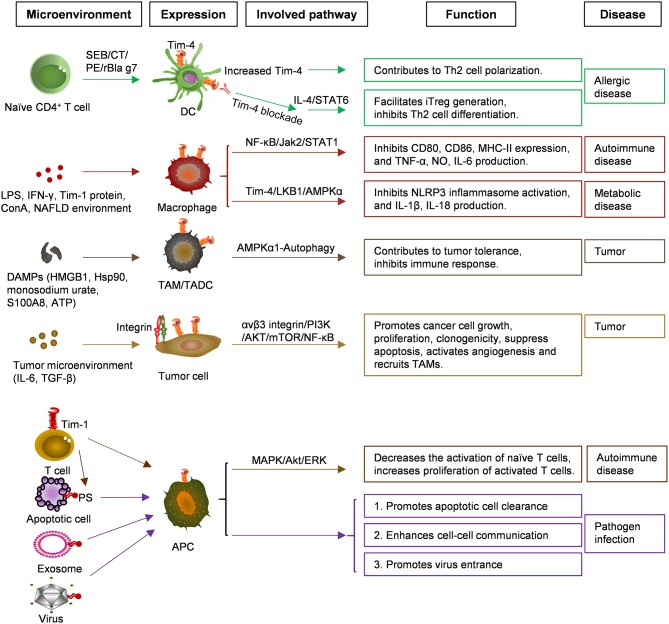
Functions and related signaling pathways of Tim-4 in various cells and diseases. Microenvironments of tumor and NAFLD enhanced Tim-4 expression in APC, TAM, TADC, tumor cells greatly, and expression of Tim-4 in APC, bound to surface PS of apoptotic cell, exosome, virus to induce signaling communication. NAFLD, Non-alcoholic fatty liver disease; APC, Antigen-presenting cell; TAM/DC, Tumor associated macrophage/dendritic cell; PS, Phosphatidylserine; SEB, Staphylococcal enterotoxin B; CT, Cholera toxin; PE, Peanut extract.

It was initially thought that Tim-4 was expressed only on APCs. However, in recent years, emerging evidence shows that Tim-4 can also be expressed in other cells, such as iNKT cells, even in tumor cells, which provided a broad scope for Tim-4 research. Under stresses or DAMPs, Tim-4 is potentially induced in epithelial cells or other cell types to promote pathology. The analysis of the Tim-4 crystal structure and the identification of its ligands provides a new pathway studying the role of Tim-4 in human diseases. Many findings show that the phagocytosis of apoptotic cells mediated by Tim-4 might play an important role in autoimmune diseases. Therefore, Tim-4 shows versatility in health and diseases (Figure 1). However, the exact role of Tim-4 in diseases remains unclear, and future research would be expected to provide novel strategies for clinical prediction and treatment.

## Author Contributions

WL and LX wrote and revised sections of the manuscript. XLia, XLiu, and YZ collected the related papers, helped to draft, and revise the manuscript. CM participated in the design of the manuscript. LG designed the manuscript and was the major contributor. All authors read and approved the final manuscript.

### Conflict of Interest

The authors declare that the research was conducted in the absence of any commercial or financial relationships that could be construed as a potential conflict of interest.
